# How hitchhiking by fire ants, *Solenopsis invicta* (Hymenoptera: Formicidae), challenges efforts for their eradication in Australia

**DOI:** 10.1093/jee/toag205

**Published:** 2026-07-11

**Authors:** Ross Wylie, Jonathan Wills, Melinda K McNaught, Erin Wallace

**Affiliations:** Department of Primary Industries, National Fire Ant Eradication Program, Berrinba, Australia; Department of Primary Industries, National Fire Ant Eradication Program, Berrinba, Australia; Department of Primary Industries, National Fire Ant Eradication Program, Berrinba, Australia; Department of Primary Industries, National Fire Ant Eradication Program, Berrinba, Australia

**Keywords:** human-mediated dispersal, invasive ants, spread pathways, pest risk mitigation

## Abstract

The ability of the red imported fire ant, *Solenopsis invicta* Buren, to spread naturally by flight or by budding from existing colonies, as well as via transport of infested goods and materials, makes containing and eradicating this species particularly challenging. In Australia, a national Program for the eradication of *S. invicta* has been in operation since its first discovery in Queensland’s capital city, Brisbane, in 2001. Since that time, 8 of the 9 known incursions of *S. invict*a into Australia have been successfully eradicated, but the main Brisbane population persists and now covers more than 830,000 hectares in urban and rural areas. As the infestation footprint has expanded, encompassing an increasing number and variety of business activities, so too has the difficulty of devising and instituting effective movement controls on products that may harbor the ant. Here, we discuss the risks and challenges posed by some of these products along with examples of dispersal events that have occurred over the life of the Program. Knowledge of *S. invicta’*s biology and behavior is key to the development of effective risk mitigation strategies.

## Graphical Abstract

**Graphical Abstract:**
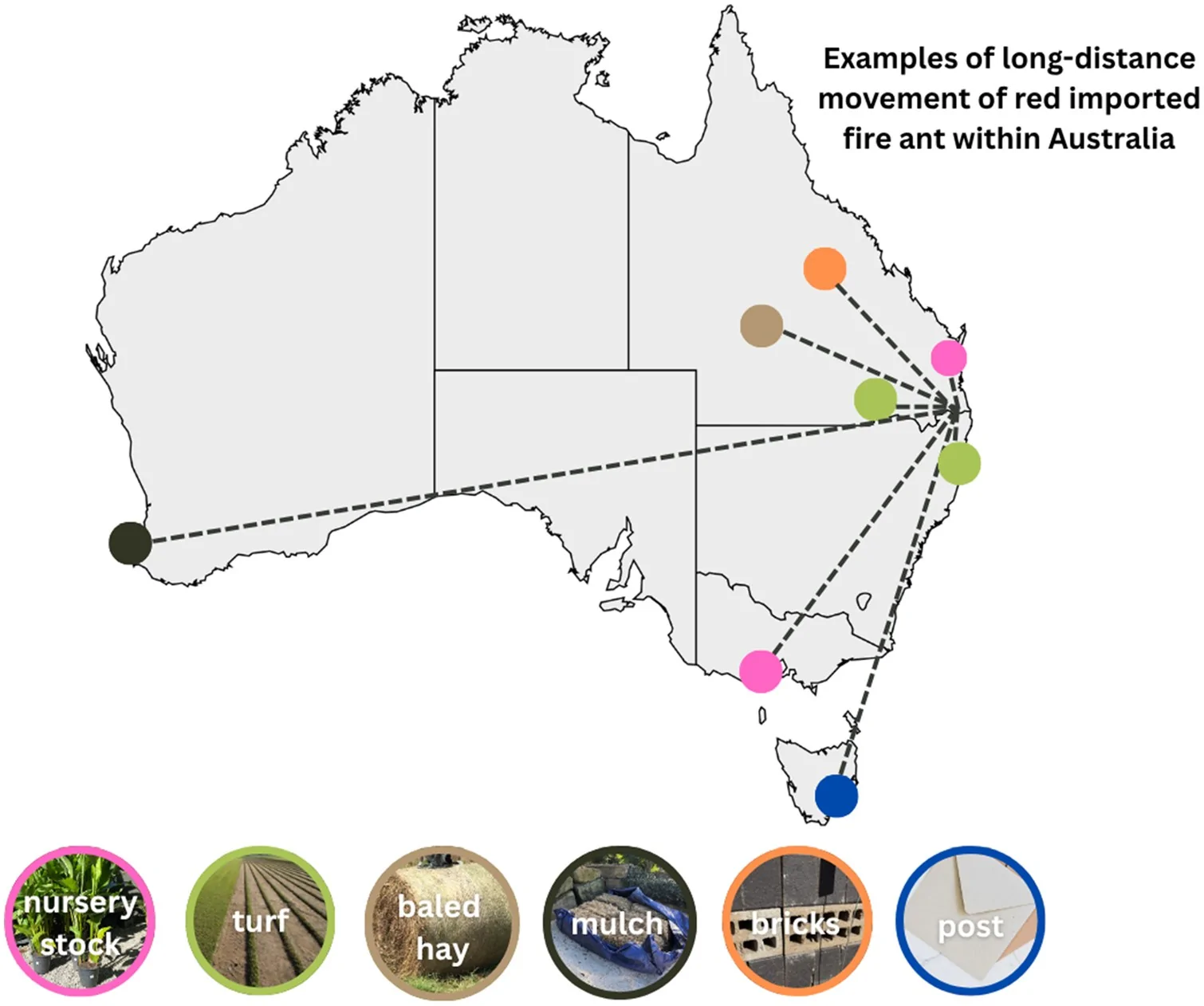

## Introduction

Social insects are among the worst invaders worldwide, and ants feature prominently in global rankings of invasives (Lowe et al. 2004, Bertelsmeier 2021). According to a recent study (Wong et al. 2023), at least 520 ant species have been transported outside of their native range. This figure greatly exceeds previous estimations of 252 (Bertelsmeier 2021) and 197 species (McGlynn 1999). Of these 520 species, 60% naturalize; others establish only indoors or not at all (Wong et al. 2023). The role of human activities in assisting this ant diaspora is well documented (Suarez et al. 2005, Gippet et al. 2019, Lach 2021).

Among 19 species generally considered as truly invasive—establishing beyond human habitation, and having direct economic, social, and environmental impacts—*Solenopsis invicta* generally ranks highest (eg. Gruber et al. 2021, Angulo et al. 2022). The first detections of *S. invicta* in Australia were in 2001 at the Port of Brisbane, Queensland and at Richlands, in the west of Brisbane (Wylie et al. 2016). The National Fire Ant Eradication Program (NFAEP, hereafter the Program) has been in operation since that time. There have been 7 subsequent incursions of this ant into Australia, all shown by genetic analysis to be new incursions. Eight of the nine known incursions have now been eradicated, including the original Port of Brisbane incursion, but the main Brisbane infestation—now covering approximately 830,000 hectares—persists, as does the effort to eradicate it.

Both social forms of *S. invicta*, monogyne (single queen) and polygyne (multiple queens), are present in the southeast Queensland population (Wylie et al. 2016). Monogyne colonies spread predominantly by nuptial flight from their parent colonies; most newly mated queen alates fly less than 2 km but in some instances can fly up to 5 km, or more with wind assistance (Vogt et al. 2000, Gruber et al. 2021, Wylie et al. 2021). Polygyne colonies mostly spread short distances by budding off a new colony fragment containing a viable queen and a cohort of workers from the parent colony. Generally, polygyne colonies with their multiple queens—nearly 700 have been recorded in a single mound in the United States (Glancey et al. 1975)—are considered of greater risk of spread by human-mediated dispersal in soil and other carriers, as well as by flooding (Wylie et al. 2024). Nevertheless, in Australia, the Program has recorded the spread of both social forms via human-mediated dispersal.

According to Gippet et al. (2019), human activities that disperse and relocate insects beyond their native ranges can be classified into 3 types of pathways: contamination, hitchhiking, and harvesting. The contamination pathway corresponds to a commodity contaminated by insects either because the commodity is the insect’s natural host or its immediate environment (eg. spread of pine woolly adelgid in Africa in infested nursery stock, Zwolinski 1989). In the hitchhiking pathway, insects attach to an object not directly related to their natural environment (eg. spread of gypsy moth in north America via eggs laid on cars, trucks, or cargo, McFadden and McMannus 1991). The harvesting pathway leads to introductions either by intentional release or subsequent escape from captivity (eg. introduction of East African lowland honey bee into Brazil to improve honey production and subsequent spread of hybrid ‘killer bees’ throughout the Americas, Winston 1992).

For *S. invicta*, human-mediated dispersal can occur via the contamination pathway, such as transport of infested soil from one location to another. It can also be dispersed via the hitchhiking pathway, for example transport in shipping containers and vehicles (Collins et al. 1993, Wylie et al. 2020). Some at-risk carriers can become infested with *S. invicta* via flights of newly mated queens rather than from already established colonies. According to Tschinkel (2006), “almost anything that moves can provide a ride for fire ants,” and he lists 86 different products or items in which *S. invicta* has been intercepted at the California Department of Food and Agriculture inspection stations (Tschinkel 2006). For this reason, combined with what we have observed as part of the Australian Eradication Program, we consider *S. invicta* to be primarily a hitchhiking species.

Key components of any pest eradication Program are the capability and capacity to detect, to kill, and to contain the pest. For *S. invicta*, each of these components has its own challenges, mainly associated with climatic variables and/or *S. invicta* behavior. For example, mound building by *S. invicta* is greatly reduced during high summer temperatures making visual detection more difficult. Heavy rainfall can affect the efficacy of baits or delay the timing of critical treatment rounds. The ability of alate reproductives of *S. invicta* to fly long distances makes containing natural spread a particular challenge which the Program addresses by multiple broadscale treatments with insect growth regulator baits over wide treatment bands (currently 5 to 10 km) beyond the observed extent of infestation, taking advantage of *S. invicta’*s documented flight capabilities and spread habits. This strategy has had demonstrated success, both at scale (Wylie et al. 2016, Wylie and McNaught 2019) and as part of “outbreak scenario” responses for containment and management (NFAEP unpublished data). However, human-mediated dispersal can undermine these containment efforts and spread the pest well beyond operational boundaries, thereby undermining eradication success.

Risk mitigation strategies are critical to reducing the risk of long distance movement that may result in entry and establishment of *S. invicta* into new environments, and to limit the scale and magnitude of impacts (EFSA Panel on Plant Health 2023, Hussain et al. 2025). Ideally, evidence of high risk carriers and dispersal pathways would be collected and recorded scientifically. The nature of invasive pest biosecurity, however, is that many detections are made incidentally by those with limited formal training and/or experience in pest biosecurity (Epanchin-Niell et al. 2021), limiting the quality and quantity of data collected. Indeed, research on initial dispersal is an understudied component of the invasion process, despite understanding initial dispersal arguably being of greatest importance to successful eradication (Puth and Post 2005). While anecdotal data may indicate a potential dispersal pathway, the credibility of such data may be called into question, particularly in instances where corroborative data is not well reflected in the peer-reviewed literature, or other credible source materials (see Leach et al. 2021). As such, the lack of reliable data describing dispersal pathways can complicate risk analysis efforts, where credibility of data is a foundational element (Schreider et al. 2010), increasing the likelihood of risk analysts inadvertently underestimating actual risk in practice. In this paper we discuss the eradication Program’s experience with the anthropogenic movement and dispersal of *S. invicta* in Australia and provide examples. Some of the difficulties for the Program in developing effective risk mitigation measures are also outlined.

## The Historic Global Spread of *S. invicta* and Suspected Carriers

Hussain et al. (2025) summarized the current global invasion status of *S. invicta*. Here, we highlight the role that known or suspected carriers play in the spread of the ant into new regions.

### The Americas

*S. invicta* has developed a reputation as an inveterate traveler, particularly in recent years. Its first recorded international foray from its native South America was to Mobile, Alabama in the southern United States in 1933 (Buren et al. 1974). It travelled by cargo ship, most likely in soil ballast although the exact carrier was never determined. In the United States, beginning after the Second World War, and in conjunction with the housing boom of the period, the spread of *S. invicta* was largely due to the movement of turf and woody ornamental plants used in landscaping (Lockley 1996). By 1953, the ant had spread to 10 southern States in the United States, and quarantine restrictions on the interstate movement of fire ant carriers were introduced in 1957. Today, *S. invicta* infests over 140 million hectares over 15 US States (Tang et al. 2025).

Starting about 1980, *S. invicta* began spreading through the West Indies, with the earliest records from Puerto Rico and the US Virgin Islands (Wetterer 2013). It is now known from 28 West Indian islands. Importation of turf from Florida, notably for the establishment of golf courses, is believed to be an important mode of spreading *S. invicta* in the West Indies (Wetterer 2013).

Although *S. invicta* had been found in Texas counties bordering the Rio Grande River in 1991, it was not until 2004 that the ant was discovered on the Mexican side of the river (Sanchez-Peña et al. 2009). Given that some colonies in Texas were on the bank of the river, spread could have been by natural flight, human-mediated dispersal, or by rafting.

### Asia

In Taiwan, the first confirmed cases of *S. invicta* were from Taoyuan City in 2003 and then in Chiayi County in 2004, with the original port of entry believed to be at or near Taoyuan International Airport (Chen et al. 2006; Yang et al. 2009). Ascunce et al. (2011) concluded that the Taoyuan infestation originated from the southern United States, and Chiayi from California. In 2014, a new infestation was discovered in Grand Kinmen Island which lies just a few kilometers east of the Chinese port city of Xiamen, and in 2018 in a container yard at the seaport of Kaohsiung; genetic analysis indicated that China was the origin of both infestations (Yang et al. 2009, Wylie et al. 2020).

In China, *S. invicta* was confirmed in Wuchuan in 2004, and Yang et al. (2012) placed the immediate source of the ants to be somewhere near Texas, United States. Long-distance spread of the ant within China was attributed to transport via nursery stock, waste plastics, tyres, turf, and animal fodder (Wang et al. 2013, Zhang and Hou 2014). Currently, *S. invicta* has been detected in 542 counties in 13 provinces (Wang et al. 2023). In 232 counties, the mode of spread was attributed to turf and plant seedlings.

In Japan, *S. invicta* was first discovered in Amagasaki in 2017 in a shipping container that originated from Guangzhou, China (Igarashi 2018). By the end of July 2019, 42 independent incursions had been detected in 14 prefectures, all except one being associated with containers from China. The exception was a detection of *S. invicta* at Narita International Airport near Tokyo in airfreight that arrived from Texas, United States in August 2018 (Hashimoto et al. 2019, 2025).

Also in 2017, fire ants were discovered in South Korea at Busan’s Gamman Port (Lyu et al. 2017). Since that time, *S. invicta* has been found at container terminals at 2 other ports associated with cargo containers shipped from China (Shim 2018). In 2018, an infestation was found at a construction site in the inland city of Daegu, in landscape rocks believed to have been imported from Guangzhou, China to decorate the grounds of an apartment complex (Shim 2018). As discussed by Wylie et al. (2020), there is believed to be at least an incipient population of *S. invicta* at 2 of India’s ports of entry, Mumbai and Kolkata.

### Oceania

As of 2025, there have been 9 genetically distinct incursions of *S. invicta* into Australia (Table 1). The first official detections were 2 separate incursions of *S. invicta* into Brisbane, Australia in February 2001: the Port of Brisbane incursion was associated with a shipping container from Florida, USA, while the south-west incursion was considered to be via a consignment of ceramic pots from Texas, USA (Wylie et al. 2016, Wylie and McNaught 2019). Of the 7 subsequent incursions, 3 have been from the United States, 3 from Argentina, and 1 from China. Six of those incursions were at ports of entry and associated with the transport of infested containers, but the exact carriers are unknown; 1 is believed to have been construction equipment and another the external motor housing of a refrigerated container. Other quarantine interceptions of *S. invicta* in Australia which did not result in establishment include a shipment of refrigerators from China, mining equipment from Texas, United States, and a vehicle from India.

**Table 1.** List of known incursions and postquarantine detections of *S. invicta* into Australia

Year	Detection	Likely country of origin	Suspected carrier/s	Location	Status
**2001**	Incursion	United States	Shipping container/s (goods unknown)	Port of Brisbane, Queensland	Last nest found Feb 2005; declared eradicated in 2012
**2001**	Incursion	United States	Shipment of ceramic pots	Richlands, Brisbane, Queensland	Eradication in progress
**2004**	Postquarantine detection	Unknown	Unknown	Port of Brisbane, Queensland	Destroyed
**2006**	Incursion	Argentina	Shipping container/s (goods unknown)	Yarwun, Queensland	Last nest found Sept 2006; declared eradicated in 2010
**2009**	Postquarantine detection	United States	Shipping container/s (goods unknown)	Lytton, Brisbane, Queensland	Destroyed
**2011**	Postquarantine detection	United States	Mining equipment	Roma, Queensland	Destroyed
**2013**	Incursion	United States	Shipping container/s (goods unknown)	Port of Gladstone, Queensland	Last nest found Sept 2014; declared eradicated in 2016
**2014**	Incursion	Argentina	Refrigerated container	Port Botany, New South Wales	Last nest found Dec 2014; area freedom declared 2016
**2015**	Incursion	United States	Shipping container/s (goods unknown)	Brisbane Airport, Queensland	Last nest found Sept 2015; declared eradicated in 2019
**2015**	Postquarantine detection	China	Shipment of refrigerators	Melbourne, Victoria	Destroyed
**2016**	Incursion	Argentina	Shipping container/s (goods unknown)	Port of Brisbane, Queensland	Last nest found May 2016; declared eradicated 2019
**2017**	Postquarantine detection	India	Imported vehicle	Adelaide, South Australia	Destroyed
**2019**	Incursion	China	Shipping container/s (goods unknown)	Port Freemantle, Western Australia	Last nest found 2019; declared eradicated in 2022
**2021**	Incursion	United States	Shipping container/s (goods unknown)	Port of Brisbane, Queensland	Declared eradicated in 2024
**2025**	Postquarantine detection	China	Shipping container/s (goods unknown)	Sydney, New South Wales	Destroyed

New Zealand has had 3 incursions of *S. invicta*: Auckland International Airport in 2001, Port of Napier in 2004, and at Whirinaki in 2006 (Christian 2009). The exact mode of entry is unknown, but all 3 *S. invicta* detections have been destroyed.

## Risk Profile Summaries for High-Risk Carriers for *S. invicta*

In Australia, *S. invicta* is listed in the National Priority List of Exotic Environmental Pests, Weeds and Diseases and is managed under the Commonwealth’s National Environmental Biosecurity Response Agreement (DAFF 2025). It is also a key threatening process to Australia’s biodiversity under the *Environment Protection and Biodiversity Conservation Act 1999* (EPBC) Act and is regulated under various State biosecurity laws. In Queensland, it is classified as Category 1 restricted matter under the *Biosecurity Act 2014* (Qld). As such, there are guidelines and regulations that place biosecurity obligations (the general biosecurity obligation) on both individuals and companies to take all reasonable and practical measures to minimize or prevent the spread of fire ants (Queensland Government 2025). This places the onus on individuals and companies to discharge their general biosecurity obligation not to move live *S. invicta* ants or nests, to report any suspected occurrence of the ant, and to comply with movement restrictions in accordance with the *Biosecurity Regulation 2016* (Qld). Failure to do so may result in financial penalties for noncompliance, or a prohibition on the movement of the product. Broadly, a fire ant carrier is any animal or plant, or part of an animal or plant, or any other thing capable of moving biosecurity matter from one place to another. Individual profiles for some of the higher risk products and mitigation challenges are discussed in more detail below.

### Risk of *S. invicta* Dispersal via Soil and Mitigation Challenges

In a hierarchy of potential carriers for spreading *S. invicta*, soil ranks as the highest, because (i) it is the insect’s natural habitat, (ii) it is a product with a high frequency and volume of transport in everyday commerce, and (iii) mitigating the risk associated with bulk soil transport is difficult.

There are numerous reports internationally of human-mediated dispersal of *S. invicta* with soil, for example, in China (Siddiqui et al. 2024), in Taiwan (Lin et al. 2021), and in the United States (Hung and Vinson 1978). All that is needed for *S. invicta* to establish a new colony at the destination is either the transport of a mated queen from an existing colony along with 10 to 25 of her workers (Tschinkel 2006), or a new queen in her claustral (egg laying) chamber, or a newly mated alate queen yet to commence egg laying. In Queensland, soil movement associated with the establishment of housing and industrial estates, roadworks and landscaping has been implicated in multiple new detections of *S. invicta* beyond biosecurity zone boundaries.

Clearly, larger scale movements and transport of soil from infested areas, whether from stockpiles or from soil excavations, represent a significant risk of accidental dispersal but even small quantities of soil harboring a viable colony have potential for relocation of the pest. Generally, soil is a high volume/low value product which makes mitigating measures such as fumigation or treatment with an insecticide financially infeasible in most cases. As discussed in more detail later in this paper, visual inspection provides no guarantee that a product is free of *S. invicta*. For soil moving within a biosecurity zone, the Program has a range of measures that apply both to pre-excavation and to the safe storage of soil, and requires vigorous mechanical disturbance (eg. screening, crushing, turning) just prior to movement (Beckett et al. 2020) (see Table 2). While the intent of the disturbance is clear *viz*., to separate the queen from her workers or to disrupt a claustral nest, what constitutes “vigorous” disturbance is somewhat subjective and the degree of efficacy has not been scientifically demonstrated. In addition, this method may not be as effective for polygyne colonies, given the increased chance of a viable colony fragment (ie. a queen and a cohort of workers) surviving the disturbance. For these reasons, soil remains a high-risk product and the movement of soil beyond biosecurity boundaries is generally prohibited.

**Table 2.** Summary of risk assessments for each carrier type and the mitigation measures currently in place in southeast Queensland. Risk estimates for each carrier taken from Virtue (2025)

Carrier	Unrestricted risk estimate	Mitigation measures
**Unprocessed soil**	Extreme	Rigorous mechanical disturbance (eg. screening, crushing, turning) just prior to movement.
**Nursery stock and potted plants (container plant production and retail nurseries)**	Low	Storage of products with overhead covering as well as mechanical or chemical barrier/s, plus chemical treatment of soil and growing media (which can include dipping, drenching and spraying or incorporating insecticide granules).
**Baled hay and baled plant materials**	Moderate	Removal of bales from the field immediately after baling, plus safe storage (covered and off the ground) or fumigation of bales where economically feasible.
**Organic mulch, compost, animal manure (bulk loose materials/stockpiles)**	Moderate	Products stored covered and off the ground or on a fire ant resistant surface, plus application of an appropriate chemical barrier and perimeter treatment, along with regular mechanical disturbance of stockpiles.
**Turf**	Extreme	Chemical treatment of turf prior to harvesting.
**Nontraditional carriers (machinery involved in earthmoving or agricultural pursuits where contamination possible)**	Moderate	Washdown/brushdown decontamination of equipment where soil or contaminants are present.

### Risk of *S. invicta* Dispersal via Nursery Stock and Potted Plants and Mitigation Challenges

The role of potted plants as a carrier and mechanism for the dispersal of a wide range of insect pests and fungal diseases is well documented, both internationally and domestically (eg. see Suarez et al. 2005, Migliorini et al. 2015), and this includes invasive ant species. A study by Suhr et al. (2019) examined 14,140 interception records (1986 to 2010) at Australian ports and found that the most common vectors for invasive ants were: live trees, plants and cut flowers; wood and wood products; edible vegetables; and edible fruits and nuts. Most interceptions included live ants (90%); castes were unknown for 86.6% of interceptions, but those interceptions for which they were recorded included a variety of forms including workers, and alate (winged) and dealate queens.

Similarly, a review of Australian interception records for tramp ants (2000 to 2015) showed that of the 37 ant species that have entered Australia, 14—including the tropical fire ant, *Solenopsis geminata* (Fabricius, 1804)—have been found in imports of nursery stock (NFAEP unpublished data). As such, this carrier represents a risk for human-mediated dispersal for *S. invicta*.

The national Program’s first experience with the long-distance movement of *S. invicta* in nursery stock was in 2001 when tracing from an infested commercial premise resulted in the detection of a small infestation near Cooroy, some 150 km north of Brisbane (associated with advanced nursery stock that contained viable colonies), and a consignment of infested plants in Victoria, more than 1350 km south of Brisbane (Vanderwoude et al. 2003). Both infestations were destroyed before they could establish (Wylie et al. 2016). In 2023, a live queen alate was found loose among a consignment of potted plants sent to Melbourne, Victoria from a nursery in Queensland. In 2024, a single worker *S. invicta* was discovered in plant material sent from Queensland to Tasmania via the postal service.

Between 2007 and 2023, 53 of the 455 nurseries (almost 12%) within the known infested area have had *S. invicta* infestation recorded on their property (NFAEP unpublished data). This is likely an underestimation of the total number since 2001, due to incomplete data pre-2007. At several nurseries, infestations have been detected in potted plants, and at others, *S. invicta* nests were located near potted stock stored at ground level. Some of these nurseries have experienced infestation on several occasions.

The principal measures that apply to the safe movement of nursery stock out of biosecurity zones are storage of the stock in a manner that prevents infestation by *S. invicta* prior to movement, or chemical treatment of associated soil/growing media (dipping, spraying or drenching of potted stock or incorporating insecticide granules in growing media) (see Table 2). Bare-rooted plants are not considered a high-risk material and can be moved without further treatment. Safe storage entails mechanical or chemical barriers to prevent incursion from ground colonies of *S. invict*a, as well as overhead covering to prevent incursions from alate (winged) mated reproductive *S. invicta* queens. Seedling plants in concrete floored, elevated bench, and covered production areas of nurseries are at little risk of infestation, but stock placed in open areas to ‘sun-harden’ before sale, sometimes for several months, are potentially at risk of infestation by alate queens although the magnitude of such risk is yet to be quantified (Virtue 2025). In the United States, female alates have been observed congregating under pots in a Florida nursery and entering the drain holes at the bottom of pots, a behavior consistent with their reported habit of finding harborage when seeking a place to establish a new nest (David Oi pers. comm.). For stock stored without cover, chemical treatment of the containerized soil/growing media provides effective protection. Unlike many other potential carriers of *S. invicta*, such chemical treatment is considered financially affordable, estimated by industry sources at approximately A$0.01 per 2L volume pot (Greenlife Queensland pers comm.). Note that this is the chemical cost only and does not include other costs such as labor and administration.

### Risk of *S. invicta* Dispersal via Baled Hay and Similar Baled Plant Materials and Mitigation Challenges

As a carrier, baled hay presents a risk of movement and translocation of ants given that it provides colonies with shelter, temperature regulation, and a potential food source. In the United States, bales left in the field and therefore in contact with the soil can become infested with *S. invicta* extremely quickly—within 1 d of being baled (Weeks et al. 2001, Drees et al. 2013). Cut and baled hay may also be infested by airborne mated female alates (Beckett et al. 2020).

There are numerous records of hay being infested with *S. invicta* in the United States (Poolaw 2017), but relatively few proven instances to date in Australia. In 2019, a consignment of baled hay destined for drought affected areas of Queensland, was intercepted before delivery and found to be infested with *S. invict*a, confirmed by an odor detection dog (Queensland Country Life 2019). In 2020, live fire ant workers and alates were documented to have been moved out of Queensland’s biosecurity zone in bales of sugarcane mulch. The movement was reported to the national program within 24 h of the material arriving at the receiving location and swift action was taken that prevented establishment or further spread. A similar movement in sugar cane mulch occurred in 2021. Given that hay is used primarily as animal feed, chemically treating the bales to remove or prevent infestation may not be feasible or desirable, except for baled products intended for mulch. Fumigation is effective but, in most cases, logistically difficult and expensive for this product. As such, removing bales from fields immediately after baling, and storage in a covered, off-ground location is the most feasible method to help prevent *S. invicta* infestation (see Table 2). Hay producers within Queensland’s restricted area must comply with regulations for the safe harvesting and storage of hay, otherwise the product is prohibited from leaving the property. Producers under quarantine or with inadequate storage facilities may be prevented from maximizing the price they receive and face increased costs of production. Moreover, quarantine restrictions around movement of baled hay out of the restricted area also impact the supply chain and can have severe implications for primary producers during drought.

### Risk of Dispersal via Organic Mulch, Compost, and Animal Manure and Mitigation Challenges

Organic mulch or composted growing media contains a wide range of products, from simple composted bark chip mulches to highly processed growing media. While these products are not likely to be infested at the point of production, stockpiles of final product will be attractive to a migrating queen and workers, or to a mated female alate—particularly where the density of fire ant nests is high (Beckett et al. 2020). Bagged products are considered a substantially lower risk than bulk products because the mechanized bagging process is likely to disrupt most nests and separate most queens from their workers (Beckett et al. 2020).

In the United States, anecdotal observations suggest that decorative mulches around ornamental plantings near homes and public areas may reduce activity of *S. invicta* or may be repellent to colonies. Thorvilson and Rudd (2001) tested finely ground, commercially available mulches, both dry and moist, of red cedar, pine bark, red cypress, cypress, and hardwood bark for repellency against laboratory and field colonies of *S. invicta*. Red cedar mulch showed some repellency; however, in general, small quantities of the mulches were not deterrent to *S. invicta* as measured by recruitment of foragers to food sources. The authors cautioned that the mulches used in the experiment were highly modified and not representative of landscaping use. In Tennessee, Long (2003) recommends limiting the use of mulch in gardens as fire ants are attracted to compost piles and mulch for warmth and moisture. In Queensland, the Program has recorded numerous instances of *S. invicta* nests in gardens or around street tree plantings landscaped with organic mulch. Nests have also been found in stockpiles, usually around the cooler edges of the pile.

Summerlin et al. (1984) studied the frequency and intensity with which *S. invicta* invaded fresh cattle droppings in Texas, USA. A total of 465 droppings were collected and 307 were found to be infested with varying numbers of fire ants. Observations of cattle droppings revealed that *S. invicta* was an active predator of larvae of the horn fly, *Haematobia irritans* (L.), and eggs and larvae of another fly, *Orthellia caesarion* (Meigen). The ants were observed invading cattle droppings within 10 min of defecation. Hu and Frank (1996) observed the formation of *S. invicta* colonies in cowpats within a week after the pats were deposited. Apart from fly larvae, these ants also reduced the populations of staphylinid, hydrophilid and carabid beetles that are predators of horn flies. In Queensland, *S. invicta* was frequently found in “cow pats” during “ground-truthing” for the remote sensing surveillance project which used thermal imaging as a primary detection tool (NFAEP unpublished data, Wylie et al. 2021) and has been found in stockpiles of cow manure (NFAEP unpublished data). Fire ants are known to forage in, and nest in, poultry houses and can infest poultry litter, which is a mixture of poultry excreta, feathers, broken eggs, spilled feed, sawdust, wood shavings (or other absorbent carbonaceous material), and is host to numerous arthropod species which are a food source for *S. invicta* (Tomberlin and Drees 2007). In a small laboratory trial in Queensland, *S. invicta* were found to nest in damp poultry litter (NFAEP unpublished data) and poultry litter was implicated as the carrier material that gave rise to an outlier detection in 2021 (NFAEP unpublished data).

Stockpiles of mulch, compost and animal manure are at risk of *S. invicta* infestation even if their core retains an inhibitory temperature because the cooler edges of the pile will rapidly become suitable nesting substrate (Beckett et al. 2020). Mitigating measures generally entail covering a stockpile in a way that prevents *S. invicta* from infesting it (eg. in a shed or under a shade cloth or tarpaulin), and either keeping it off the ground or at ground level on a fire ant resistant surface with an appropriate chemical barrier and perimeter treatment (see Table 2). Mitigation challenges include a difficulty in adequately covering large stockpiles and in maintaining chemical barriers. For these reasons, regular mechanical disturbance of stockpiles is the approach generally adopted, the aim being to disrupt any fire ant colonies that may have established, separating the queen from her workers ensuring her demise, or to disrupt a queen and brood within her claustral chamber (Beckett et al. 2020).

### Risk of Dispersal via Turf and Mitigation Challenges

The cycle of production on turf farms creates an environment that is inherently attractive to fire ants, *viz*., open, sunny, and grassed areas that are mown, watered and fertilized, as well as disturbed ground that remains bare postarvest until the next crop comes through. Australian turf is lifted with approximately 2 to 3 cm of soil and, in principle, may include a fire ant queen and enough workers, a new queen in an incipient nest, or a recently landed mated female alate (Beckett et al. 2020). Chemical treatment of turf prior to harvesting, as required under the regulation, reduces the likelihood that a fire ant queen or incipient nest is moved with the turf (Table 2). There is, however, a risk that a recently mated female alate that has not commenced nesting may land on rolls or squares of harvested turf prior to dispatch, thereby avoiding areas with higher concentrations of insecticide (superficial thatch and the top few millimeters of soil). A recent detection in New South Wales was the first time a newly mated queen had been found directly in the turf rolls before the turf was laid (Pauline Lenancker pers. comm.). Because harvested turf cannot be stored for more than 48 h before it begins to deteriorate, it is not as vulnerable to postharvest infestation as most other fire ant carriers. A failure in compliance or accidental errors in the application of the chemical treatment have the potential to result in a significant infestation and a significantly high-risk carrier (Beckett et al. 2020).

The attraction of turf farm environments to mated female alates on nuptial flights was corroborated in an investigation in 2017 by the Program into the apparent persistence of fire ant infestations on turf farms. Microsatellite analysis was used to determine relationships between *S. invicta* nests found on farms in the study over a 3- to 5-yr period. Results showed that nests on the 8 farms included in the study were mostly monogyne, with microsatellite analysis recording a series of direct or indirect relationships between sampled nests. Importantly, in this analysis, there were no recorded instances of nest survival during the study period (NFAEP unpublished data). These results suggested that the persistent infestation was not the result of survivors of chemical treatment but rather new infestations from mated female alates produced from nests located on adjacent land. It also showed a tendency for alates from a parent nest on, or proximal to, a turf farm to recolonize other parts of the same site.

### Risk of Dispersal via Items Not Classified as Fire Ant Carriers

The United States experience with movement of fire ants in a range of unlikely products (Tschinkel 2006) is reflected in Australia, most recently in 2025 with a long-distance movement (approximately 1000 km) of *S. invicta* via a consignment of palletized bricks from an infested area of Brisbane to a mine site in central Queensland. There the ants had established nests, prompting a major eradication response from the Program. Machinery and vehicles, of themselves, are not classified as fire ant carriers except where they have been actively involved in earthmoving or agricultural pursuits where potentially contaminated product such as soil or hay may be attached. In these cases, washdown/brushdown decontamination is required (Table 2). Goods may be inadvertently infested with *S. invicta* when transported on pallets or in containers that have been in ground contact, usually for prolonged periods, in infested areas and in which the ants have built a nest.

## Discussion

Tschinkel (2006) observed that “mankind is the fire ant’s best friend,” alluding to the role that human-mediated dispersal has played in the spread of *S. invicta* internationally and domestically. In 2001, at the beginning of Australia’s efforts to eradicate this pest, the Program benefited greatly from the United States experience with *S. invicta* in framing a response to detection, treatment and containment. Of note was the rapid spread of *S. invicta* via product movement to 10 southern States in the US before quarantine controls were introduced in 1957. There was thus an imperative to quickly develop and implement risk mitigation measures to prevent similar spread in Australia. In developing these measures, the intent was to exploit known aspects of *S. invicta’*s behavior and biology, while also incorporating what would be both practical and economically feasible for businesses and stakeholders to comply with. Fortunately, in this regard, *S. invicta* is a relatively well studied insect, in contrast to some other highly invasive and emerging ant invaders in Australia such as the Browsing ant *Lepisiota frauenfeldi* where aspects of their biology and behavior are less understood (Hoffmann et al. 2023).

Initially in Queensland, businesses dealing with known fire ant carriers were encouraged to apply for an approved risk management plan (ARMP), administered by the Program, that allowed them to move their product freely if compliant with stipulated guidelines. A key requirement of an ARMP was that the property had been visually inspected and found to be free of fire ants (“property freedom”). While visual surveillance remains an important and affordable primary measure for detecting the presence of *S. invicta*, it cannot guarantee that a property or product is “fire ant free.” An incipient (newly established) fire ant colony may show no visible signs of its existence through soil disturbance for 3 to 5 months after founding (Tschinkel 2006). As well, in high summer temperatures the ants retreat deeper into the soil profile and their above ground mounds weather away leaving no trace of fire ant presence. Thus, property freedom based principally on visual surveillance and reactive treatment, for an ant that may be undetectable by this method for several months of a year, is insufficient to entirely mitigate the risk of movement of fire ants in any carrier. This finding has been confirmed many times and in multiple situations by the Program’s fire ant odor detection dogs, that regularly detect fire ants where there are no visible above-ground signs. ARMPs were discontinued in 2016 and the focus switched from property freedom to product freedom.

Visual surveillance also has its shortcomings for confirming product freedom. With nursery stock, for example, it is often argued by industry proponents that the multiple handling of potted plants in nursery production systems would reveal the presence of fire ants. This may be the case for an established infestation in a potted plant, but not for a newly mated queen in her claustral chamber or an incipient nest with a handful of workers. Likewise, visual inspection of stockpiles of soil, mulch or mined products, or of baled plant products and turf is likely to be ineffective. Instead, under the regulation, product freedom for fire ants generally relies on either chemical treatment, or vigorous mechanical disturbance such as grinding, crushing, shredding, tumbling, washing, or storage of product in a manner to prevent infestation. A combination of these measures can also provide greater confidence of product freedom than visual surveillance can provide alone.

Australian States and Territories vary in their movement controls/entry conditions for the movement of *S. invicta* carriers out of fire ant biosecurity zones in Queensland to other jurisdictions. This can create unnecessary complexities for businesses moving such products and a national pest risk analysis (PRA) for *S. invicta* is underway, seeking nationally harmonized risk management measures (Virtue 2025). A PRA is a formal process of assessing the likelihood and potential consequences of a pest, to determine the risk it poses and what, if any, risk management measures are needed to reduce this risk to an appropriate level of protection (ALOP), defined in Australia as being to a very low level, but not to zero (Virtue 2025). A PRA is, quite rightly, heavily evidence based but if such evidence is, for a variety of reasons, difficult to collect, does this equate to very low risk?

In Australia, there have been hundreds of instances where human-mediated dispersal of *S. invicta* in carriers has been demonstrated or implicated where *S. invicta has* been detected well beyond what would have been possible by natural dispersal. Many of these were reported to the Program by members of the public, and linked to new residential or commercial developments, roadworks, or landscaping, with possible carriers being turf, soil (both natural and manufactured), mulch, and nursery plants. The Program responds to these ‘outliers’ with surveillance, treatment, genetic analysis, and tracing activities to detect possible sources. Where multiple potential carriers are involved, it is not always possible to distinguish or exonerate individual carriers. This has resulted in relatively low numbers of “proven” instances of *S. invicta* being moved in individual carriers which greatly influences the results of a PRA. The consequences of spread of this pest in Australia are extreme, as generally acknowledged. When weighing up available options for risk mitigation measures based on likelihood/probabilities, it is instructive to consider that the current 830,000 ha infestation in southeast Queensland originated from just a few *S. invicta* queens as shown by genetics (NFAEP unpublished data).

Measures adopted by Australia to prevent or limit the spread of *S. invict*a in products have evolved over the past 24 years with increasing knowledge of the biology and behavior of the ant and the Program’s experience with fire ant carriers. For ant species in general, nesting habits play a key role in determining the probability of that species establishing in a new location. For example, an analysis of interception records of nonnative ants at ports-of-entry in the United States found that ground nesting species are more likely to establish than arboreal species (Suarez et al. 2005). They attributed this to a comparative shortage of nest sites for arboreal ants in new environments, often because of that ant species’ preferences for types of trees. Establishment is also dependent on other conditions prevailing at the destination site, such as inhospitable terrain (lack of food, water or shelter), adverse climatic conditions, and inspection or pest management regimes in place to detect or prevent successful establishment. Another consideration with human-mediated dispersal is the ability of the ant species to survive the stresses associated with the transport process. In all these regards, *S. invicta* has demonstrated its ability to survive and establish even in the harshest conditions. Australian examples include establishment at the Port of Gladstone, Queensland in a site composed of reclaimed sand and coral fragments and a “hard stand” of compressed crusher dust and gravel (Wylie et al. 2021), and long-distance transport from the United States in the external motor housing of a refrigerated container to establish at Port Botany in Sydney, New South Wales. Both incursions have since been eradicated (Wylie and McNaught 2019).

*S. invicta’*s complex reproductive strategies make it one of the most challenging of the invasive ant species to control, delimit and contain. In general, other invasive ants—in particular the yellow crazy ant (*Anoplolepis gracilipes*), the little fire ant or electric ant (*Wasmannia auropunctata*), and the Argentine ant (*Linepithema humile*)—reproduce via budding and no longer disperse via nuptial flights (Holway et al. 2002). *S. invicta*, however, has both monogyne and polygyne social forms, each with their own risks and spread pathways. Within the remaining *S. invicta* population in southeast Queensland, most colonies are monogyne, with a small proportion of the population being polygyne (Wylie et al. 2021). Monogyne colonies can have a very high production of female alates and the risk of infestation of products in the open and unprotected should not be underestimated. Despite their lower overall presence in the southeast Queensland population, polygyne *S. invicta* colonies, with their multiple queens, pose a significant risk of spread via human-mediated dispersal. Moreover, polygyne colonies can produce monogyne queen alates which increases the spread risk of any polygyne population overall. This increased risk, combined with the fact that polygyne populations tend to reach higher nest densities (Porter et al. 1991), and can take longer to treat and destroy (McNaught et al. 2014), means that any known polygyne *S. invicta* infestation should be considered a treatment priority and particular attention paid to the investigation of product movement from a polygyne site.

There are many other ways in which the Program exploits aspects of *S. invicta* biology and behavior when designing risk mitigation strategies. For example, knowledge that fire ants have critical thermal limits means that composting or heating can be used to disinfest some products prior to movement where logistically feasible. After an *S. invicta* queen’s fat reserves are exhausted she is reliant on workers for nutrition and survival, and this is the principle behind vigorous disturbance of product stockpiles before movement to separate the queen from her workers and ensure that any colony fragment is not viable. A nuptial queen’s choice of where to land to establish a new colony is not random and there appears to be a preference for disturbed land types, such as new residential or commercial developments (Tschinkel 1993, NFAEP unpublished data). Businesses moving product from such sites are required to institute a program of regular broadcast bait treatment to mitigate risk.

*S. invicta* continues its spread throughout the world, primarily via human-mediated dispersal. When compared to other countries across *S. invicta’*s introduced range, the legislated movement and storage control measures in place in Australia have played a crucial role in largely containing the ant and slowing its rate of outward spread from the southeast Queensland incursion over time (Wylie et al. 2020, Queensland Government 2025). After 24 years, and despite several new incursions and outlier detections, the ant is still contained in a relatively small area of Australia (less than a million hectares). For countries newly invaded or at risk of invasion by *S. invicta* (see Wylie et al. 2020, Gruber et al. 2021), the importance of implementing measures to reduce the risk of human-mediated dispersal of infested carriers, keeping open prospects of eradication and minimizing environmental and economic impacts, cannot be overstated.

## Data Availability

There are no new data associated with this article. Program data is available on request.

## References

[toag205-B1] Angulo E , Hoffmann BD, Ballesteros-Mejia L, et al 2022. Economic costs of invasive alien ants worldwide. Biol. Invasions 24:2041–2060. 10.1007/s10530-022-02791-w

[toag205-B2] Ascunce MS , Yang C-C, Oakey J, et al 2011. Global invasion history of the fire ant *Solenopsis invicta*. Science 331:1066–1068. 10.1126/science.1198734 21350177

[toag205-B3] Beckett S , Pettit M, Froese J, et al 2020. Review of red imported fire ant scientific principles and controls: an evaluation for the national red imported fire ant eradication program. CSIRO Health and Biosecurity.

[toag205-B4] Bertelsmeier C. 2021. Globalization and the anthropogenic spread of invasive social insects. Curr. Opin. Insect Sci. 46:16–23. 10.1016/j.cois.2021.01.006 33545436

[toag205-B5] Buren WF , Allen GE, Whitcomb WH, et al 1974. Zoogeography of the imported fire ants. J. New York Entomol. Soc. 82:113–124.

[toag205-B6] Chen JSC , Shen C-H, Lee H-J. 2006. Monogynous and polygynous red imported fire ants. Solenopsis invicta Buren (Hymenoptera: Formicidae), in Taiwan. Environ. Entomol. 35:167–172. 10.1603/0046-225X-35.1.167

[toag205-B7] Christian S. 2009. Red imported fire ants eradicated from Napier. Biosecurity 92:28–29.

[toag205-B8] Collins HL , Lockley TC, Adams DJ. 1993. Red imported fire ant (Hymenoptera: Formicidae) infestation of motorized vehicles. Fla. Entomol. 76:515–516. 10.2307/3495651

[toag205-B9] DAFF. 2025. The national priority list of exotic environmental pests, weeds and diseases - DAFF. Agriculture.gov.au. https://www.agriculture.gov.au/biosecurity-trade/policy/environmental/priority-list

[toag205-B10] Drees BM , Calixto AA, Nester PR. 2013. Integrated pest management concepts for red imported fire ants *Solenopsis invicta* (Hymenoptera: Formicidae). Insect Sci. 20:429–438. 10.1111/j.1744-7917.2012.01552.x 23955939

[toag205-B11] EFSA Panel on Plant Health, Bragard C , Baptista P, Chatzivassiliou E, et al 2023. Pest categorisation of *Solenopsis invicta*. EFSA J. 21:7998. 10.2903/j.efsa.2023.7998 PMC10205889 37234270

[toag205-B12] Epanchin-Niell R , Thompson AL, Treakle T. 2021. Public contributions to early detection of new invasive pests. Conservat. Sci. Prac. 3:e422. 10.1111/csp2.422

[toag205-B13] Gippet JM , Liebhold AM, Fenn-Moltu G, et al 2019. Human-mediated dispersal in insects. Curr. Opin. Insect Sci. 35:96–102. 10.1016/j.cois.2019.07.005 31479895

[toag205-B14] Glancey BM , Stringer CE, Craig CH, et al 1975. An extraordinary case of polygyny in the red imported fire ant. Ann. Entomol. Soc. Am. 68:922. 10.1093/aesa/68.5.922

[toag205-B15] Gruber MAM , Janssen-May S, Santoro D, et al 2021. Predicting socio-economic and biodiversity impacts of invasive species: red imported fire ant in the developing western Pacific. Eco. Manage. Restorat. 22:89–99. 10.1111/emr.12457

[toag205-B16] Hashimoto Y , Sakamoto H, Mitsuhashi H. 2025. The battle against the red imported fire ant, *Solenopsis invicta* (Hymenoptera: Formicidae), in Japan: the imminent threat of establishment and border control measures. Global Environ. Res. 28:101–107. 10.57466/ger.28.2_101

[toag205-B17] Hashimoto Y , Yoshimura M, Huang R-N. 2019. Wasabi versus red imported fire ants: preliminary test of repellency of microencapsulated allyl isothiocyanate against *Solenopsis invicta* (Hymenoptera: Formicidae) using bait traps in Taiwan. Appl. Entomol. Zool. 54:193–196. 10.1007/s13355-019-00613-5

[toag205-B18] Hoffmann B , Widmer M, Bates O. 2023. Preparing to eradicate a novel invader of unknown biology: a case study from Australia. MBI. 14:421–436. 10.3391/mbi.2023.14.3.03

[toag205-B19] Holway DA , Lach L, Suarez AV, et al 2002. The causes and consequences of ant invasions. Annu. Rev. Ecol. Syst. 33:181–233. 10.1146/annurev.ecolsys.33.010802.150444

[toag205-B20] Hu GY , Frank JH. 1996. Effect of the red imported fire ant (Hymenoptera: Formicidae) on dung-inhabiting arthropods in Florida. Environ. Entomol. 25:1290–1296. 10.1093/ee/25.6.1290

[toag205-B21] Hung ACF , Vinson SB. 1978. Factors affecting the distribution of fire ants in Texas (Myrmicinae: Formicidae). Southwest. Nat. 23:205–213. 10.2307/3669769

[toag205-B22] Hussain M , Gao X, Ijaz M, et al 2025. The red imported fire ant (*Solenopsis invicta* Buren, 1972): a persistent global invader and the search for effective control. Neotrop. Entomol. 54:135. 10.1007/s13744-025-01349-4 41436899

[toag205-B23] Igarashi K. 2018. Environment ministry taking steps in bid to prevent fire ants from settling in Japan. The Mainichi. https://mainichi.jp/english/articles/20180508/p2a/00m/0na/020000c

[toag205-B24] Lach L. 2021. Invasive ant establishment, spread, and management with changing climate. Curr. Opin. Insect Sci. 47:119–124. 10.1016/j.cois.2021.06.008 34252591

[toag205-B25] Leach LF , Baker C, Leamons CG, et al 2021. Using evidence to frame problems of practice. IE. 6:1–7. 10.5195/ie.2021.144

[toag205-B26] Lin C-H , Wen T-H, Liu Y-H, et al 2021. Elucidating how the red imported fire ant (*Solenopsis invicta*) diffused spatiotemporally among different landscapes in north Taiwan, 2008–2015. Ecol. Evol. 11:18604–18614. 10.1002/ece3.8465 PMC8717340 35003696

[toag205-B27] Lockley TC. 1996. Imported fire ants. University of Minnesota Radcliffe’s IPM World Textbook. https://ipmworld.umn.edu/lockley

[toag205-B28] Long EA. 2003. A management plan for the imported fire ant (IFA) Migration into gateway communities in east Tennessee. The University of Tennessee. https://trace.tennessee.edu/handle/20.500.14382/37976

[toag205-B29] Lowe S , Browne M, Boudjelas S, et al 2004. 100 of the world’s worst invasive alien species. A selection from the Gobal Invasive Species Database. https://www.iucngisd.org/gisd/100_worst.php

[toag205-B30] Lyu D , Pyo Lee H, Sik, et al 2017. The red imported fire ant. Solenopsis invicta Buren (Hymenoptera: Formicidae: Myrmicinae) discovered in Busan Sea Port, Korea. Korean J. Appl. Entomol. 56:437–438. 10.5656/KSAE.2017.11.0.049

[toag205-B31] McFadden MW , McMannus ME. 1991. An insect out of control? The potential for spread and establishment of the Gypsy moth in new forest areas in the United States. In: Baranchikov YN, Mattson WJ, Hain WJ, et al, editors. Forest insect guilds: patterns of interaction with host trees. US Department of Agriculture Forest Service General Technical Report. pp.172–186.

[toag205-B32] McGlynn TP. 1999. The worldwide transfer of ants: geographical distribution and ecological invasions. J. Biogeogr. 26:535–548. 10.1046/j.1365-2699.1999.00310.x

[toag205-B33] McNaught MK , Wylie R, Harris F, et al 2014. Effect of broadcast baiting on abundance patterns of red imported fire ants (Hymenoptera: Formicidae) and key local ant genera at long-term monitoring sites in Brisbane, Australia. J. Econ. Entomol. 107:1307–1315. 10.1603/EC14008 25195416

[toag205-B34] Migliorini D , Ghelardini L, Tondini E, et al 2015. The potential of symptomless potted plants for carrying invasive soilborne plant pathogens. Diversity Distrib. 21:1218–1229. 10.1111/ddi.12347

[toag205-B35] Poolaw R. 2017. Red imported fire ant is being transported in donated hay bales. https://www.kswo.com/story/34784277/red-imported-fire-ant-is-being-transported-in-donated-hay-bales/

[toag205-B36] Porter SD , Bhatkar A, Mulder R, et al 1991. Distribution and density of polygyne fire ants (Hymenoptera: Formicidae) in Texas. J. Econ. Entomol. 84:866–874. 10.1093/jee/84.3.866 1885844

[toag205-B37] Puth LM , Post DM. 2005. Studying invasion: have we missed the boat? Ecol. Lett. 8:715–721. 10.1111/j.1461-0248.2005.00774.x

[toag205-B38] Queensland Country Life. 2019. Fire ant infested hay found outside biosecurity zone. https://www.queenslandcountrylife.com.au/story/6539765/fire-ant-infested-hay-found-outside-biosecurity-zone/

[toag205-B39] Queensland Government. 2025. National fire ant eradication program: general biosecurity obligation guideline. https://www.publications.qld.gov.au/dataset/general-biosecurity-obligation-guideline

[toag205-B40] Sanchez-Peña SR , Chacón-Cardosa MC, Resendez-Perez D. 2009. Identification of fire ants (Hymenoptera: Formicidae) from northeastern Mexico with morphology and molecular markers. Fla. Entomol. 92:107–115. 10.1653/024.092.0117

[toag205-B41] Schreider J , Barrow C, Birchfield N, et al 2010. Enhancing the credibility of decisions based on scientific conclusions: transparency is imperative. Toxicol. Sci. 116:5–7. 10.1093/toxsci/kfq102 20363830

[toag205-B42] Shim K-S. 2018. Fire ant queen found in Daegu. *Korea JoongAng Daily*. https://koreajoongangdaily.joins.com/2018/09/18/socialAffairs/Fire-ant-queen-found-in-Daegu/3053371.html

[toag205-B43] Siddiqui JA , Bamisile BS, Fan R, et al 2024. Ant invasion in China: an in-depth analysis of the country’s ongoing battle with exotic ants. Ecol. Indic. 160:111811. 10.1016/j.ecolind.2024.111811

[toag205-B44] Suarez AV , Holway DA, Ward PS. 2005. The role of opportunity in the unintentional introduction of nonnative ants. Proc. Natl. Acad. Sci. USA. 102:17032–17035. 10.1073/pnas.0506119102 PMC1287985 16286656

[toag205-B45] Suhr EL , O’Dowd DJ, Suarez AV, et al 2019. Ant interceptions reveal roles of transport and commodity in identifying biosecurity risk pathways into Australia. NB. 53:1–24. 10.3897/neobiota.53.39463

[toag205-B46] Summerlin JW , Harris RL, Petersen HD. 1984. Red imported fire ant (Hymenoptera: Formicidae): frequency and intensity of invasion of fresh cattle droppings. Environ. Entomol. 13:1161–1163. 10.1093/ee/13.5.1161

[toag205-B47] Tang X , Deng Y, He Z, et al 2025. Modelling the potential distribution and niche shift of *Solenopsis invicta* Buren under climate change and invasion process. Front. For. Glob. Change 8:1659630–1659648. 10.3389/ffgc.2025.1659630

[toag205-B48] Thorvilson H , Rudd B. 2001. Are landscaping mulches repellent to red imported fire ants? Southwest. Entomol. 26:195–203.

[toag205-B49] Tomberlin JK , Drees B. 2007. Poultry pest management. Texas A&M AgriLife. https://extensionentomology.tamu.edu/resources/management-guides/poultry-pest-management/

[toag205-B50] Tschinkel WR. 1993. The fire ant (*Solenopsis* *invicta*): still unvanquished. In: McKnight B, editor. Biological pollution: the control and impact of invasive exotic species. Indiana Academy of Science, Indianapolis. p. 121−136.

[toag205-B51] Tschinkel WR. 2006. The fire ants. Belknap Press.

[toag205-B52] Vanderwoude C , Elson-Harris M, Hargreaves JR, et al 2003. An overview of the red imported fire ant (*Solenopsis invicta*) eradication plan for Australia. Records South Austr Museum Monogr Ser 7:11–16.

[toag205-B53] Virtue JG. 2025. Pest risk analysis for potential carriers of red imported fire ant (Solenopsis invicta Buren) in Australia. A report prepared for Plant Health Committee. JG Virtue Biosecurity Services Pty Ltd.

[toag205-B54] Vogt JT , Appel AG, West MS. 2000. Flight energetics and dispersal capability of the fire ant, *Solenopsis invicta* Buren. J. Insect Physiol. 46:697–707. 10.1016/S0022-1910(99)00158-4 10742518

[toag205-B55] Wang L , Lu Y, Xu Y, et al 2013. The current status of research on *Solenopsis invicta* Buren (Hymenoptera: Formicidae) in mainland China. Asian Myrmecol. 5:125–137.

[toag205-B56] Wang X , Qin Y, Xu Y, et al 2023. Surveillance and invasive risk of the red imported fire ant, *Solenopsis invicta* Buren in China. Pest Manag. Sci. 79:1342–1351. 10.1002/ps.7297 36412239

[toag205-B57] Weeks RD , Heimer ME, Barr CL, et al 2001. Evaluation of potential imported fire ant quarantine treatments for hay bales. Proceedings of the 2001 annual imported fire ant research conference, 28 February–2 March 2001, San Antonio, Texas, USA. Texas Agricultural Extension Service, Texas A&M University.

[toag205-B58] Wetterer JK. 2013. Exotic spread of *Solenopsis invicta* Buren (Hymenoptera: Formicidae) beyond North America. Sociobiology 60:50–55. 10.13102/sociobiology.v60i1.50-55

[toag205-B59] Winston ML. 1992. Killer bees: the Africanized honey bee in the Americas. Harvard University Press. https://www.degruyterbrill.com/document/doi/10.4159/harvard.9780674593954.c1/html

[toag205-B60] Wong MKL , Economo EP, Guénard B. 2023. The global spread and invasion capacities of alien ants. Curr. Biol. 33:566–571.e3. 10.1016/j.cub.2022.12.020 36610395

[toag205-B61] Wylie FR , Oakey J, Williams ER. 2021. Alleles and algorithms: the role of genetic analyses and remote sensing technology in an ant eradication program. NB. 66:55–73. 10.3897/neobiota.66.64523

[toag205-B62] Wylie R , Jennings C, McNaught MK, et al 2016. Eradication of two incursions of the red imported fire ant in Queensland, Australia. Eco. Manag. Restorat. 17:22–32. 10.1111/emr.12197

[toag205-B63] Wylie R , McKinney M, McLennan A, et al 2024. Floods and fire ants, *Solenopsis invicta* (Hymenoptera: Formicidae): the Australian experience. Austral. Entomol. 63:369–378. 10.1111/aen.12692

[toag205-B64] Wylie R , McNaught MK. 2019. Eradication of red imported fire ants in Australia (NRIFAEP Brisbane) – UPDATE to EMR feature. EMR Project Summaries. https://site.emrprojectsummaries.org/2019/09/25/eradication-of-red-imported-fire-ants-in-australia-nrifaep-brisbane-update-to-emr-feature/

[toag205-B65] Wylie R , Yang C-C, Kazuki T. 2020. Invader at the gate: the status of red imported fire ant in Australia and Asia. Ecol. Res. 35:6–16. 10.1111/1440-1703.12076

[toag205-B66] Yang C-C , Ascunce MS, Luo L-Z, et al 2012. Propagule pressure and colony organization are associated with the successful invasion and rapid range expansion of fire ants in China. Mol. Ecol. 21:817–833. 10.1111/j.1365-294X.2011.05393.x 22181975

[toag205-B67] Yang C-CS , Shoemaker DD, Wu J-C, et al 2009. Successful establishment of the invasive fire ant *Solenopsis invicta* in Taiwan: insights into interactions of alternate social forms. Divers. Distrib 15:709–719. 10.1111/j.1472-4642.2009.00577.x

[toag205-B68] Zhang X , Hou Y-M. 2014. Invasion history of *Solenopsis invicta* (Hymenoptera: Formicidae) in Fujian, China based on mitochondrial DNA and its implications in development of a control strategy. Insect Sci. 21:493–498. 10.1111/1744-7917.12042 23956039

[toag205-B69] Zwolinski JB. 1989. The pine woolly aphid, *Pineus pini* (L.)—A pest of pines in South Africa. South Afr. Forestry J. 151:52–57. 10.1080/00382167.1989.9630506

